# Melatonin nuclear receptors mediate monochromatic light-induced T-lymphocyte proliferation of thymus through the AKT/GSK3β/β-catenin pathway in chick

**DOI:** 10.1016/j.psj.2024.104507

**Published:** 2024-11-05

**Authors:** Juanjuan Xiong, Zixu Wang, Yulan Dong, Jing Cao, Yaoxing Chen

**Affiliations:** a College of Animal Science and Food Engineering, Jinling Institute of Technology, Qixia, Nanjing, 210046, China; b Laboratory of Anatomy of Domestic Animals, College of Veterinary Medicine, China Agricultural University, Haidian, Beijing, 100193, China

**Keywords:** Signal pathway, RORα/RORγ, Thymocytes, Red light, Broiler

## Abstract

Based on previous research, it's unclear about the signaling pathway involved in the negative regulation of T-lymphocyte proliferation in thymus by monochromatic red light. Newly hatched chicks were randomly assigned divided into white (**WL**), red (**RL**), green (**GL**), and blue (**BL**) light treatments. Three days later, each light treatment group was further divided into intact, sham operation, and pinealectomy groups. The findings revealed that RL led to an increase in the expression of RORα and RORγ, while p-AKT/p-GSK3β/β-catenin/CyclinD1 expression in the thymus of chicks were decreased. Conversely, GL showed opposite results compared to RL. After pinealectomy, accompanied with the expression of RORα and RORγ increased under four light, p-AKT/ p-GSK3β/ β-catenin/ CyclinD1 expression were decreased. In vitro, exogenous melatonin increased the p-AKT/β-catenin/CyclinD1 expression in the thymic lymphocytes of chick reared under RL. The stimulative effect of melatonin was enhanced by SR3335 (RORα antagonist) or GSK298 (RORγ antagonist), while it was attenuated by SR1078 (RORα/RORγ agonist), LY-294 (PI3K antagonist) and HY-102 (AKT antagonist). These results demonstrate that RORα/RORγ negatively regulate monochromatic red light induced-T-lymphocyte proliferation in the thymus, possibly through the PI3K/AKT/p-GSK3β (Ser9) signaling pathway.

## Introduction

The avian retina possesses one of the most sophisticated photoreceptor systems among vertebrates (Kram et al., 2010), therefore birds have a very sensitive perception of light. Numerous studies have demonstrated the impact of light on immune function in both mammals and birds. Continuous darkness can cause pathological hypertrophy of the thymus in rats, while continuous light can induce thymus degeneration and lymphocyte death (Mahmoud et al., 1994). Besides, changes in the expression of natural regulatory T-cells in the thymus may be involved in the immune system's response to day and night cycles (Kiernozek et al., 2014). Although studies have highlighted the effects of light illuminance and photoperiod on bird immune function (Lien et al., 2007), there is still a lack of research on the impact of light wavelength on chick immunity. Our previous study demonstrated that green light promotes immune function development in early-stage broilers (Zhang et al., 2014) including in the spleen (Guo et al., 2015), thymus (Chen et al., 2016) and bursa of Fabricius (Li et al., 2015b). However, the mechanism of how light color affects this process is still not comprehensive.

Numerous studies have shown that melatonin plays a crucial role in regulating the impact of light on immune function (Horodincu and Solcan, 2023). For example, the photoperiod can adjust peripheral melatonin levels to enhance immune response and improve survival in hamsters (Vishwas and Haldar, 2013). In broilers, monochromatic green light promoted the plasma melatonin levels to induce the proliferation of T- lymphocytes (Chen et al., 2016), while red light inhibited plasma melatonin levels (Xiong et al., 2019). Melatonin travels to target cells and binds to receptors to exert its regulatory effects. Studies have pointed out that, melatonin membrane receptors (**MT1/MT2**) expression in B-lymphocyte were closely related to the melatonin levels, and were regulated by light in mice (Cernysiov et al., 2014). Similar to what we found in our previous study, melatonin combines with the membrane receptors 1b (**Mel1b**) and **Mel1c** mediated the monochromatic green light-induced T-lymphocyte proliferation of thymus in chick (Chen et al., 2016). On the contrary, the long wavelength (red light) showed the higher mRNA and protein levels of Mel1a and Mel1c in ovarian follicle of chicken (Li et al., 2015a). This may be due to the different expression of receptors in different tissues, and melatonin works through different receptor signals. In addition to binding to membrane receptors, melatonin also acts through nuclear receptors (Billon et al., 2016; Lardone et al., 2011). Although, there have been limited studies on nuclear receptors in chicken lymphocytes. Our previous research demonstrated that melatonin, via the nuclear receptor RORα, regulates the proliferation of thymic lymphocytes induced by monochromatic light (Xiong et al., 2019). However, the mechanism by which melatonin activates downstream signals through nuclear receptors to further regulate light-induced lymphocyte proliferation remains unclear.

Studies have shown that the Wnt signaling pathway plays a crucial role in the immune system. The canonical Wnt signaling pathway involves β-catenin/TCF-LEF signaling molecules (Jamieson et al., 2012). β-catenin binds to the cytoplasmic complexes and remains in a stable state in unstimulated cells. These complexes contain axis inhibition protein (**AXIN**), adenomatous polyposis coli, casein kinase 1 (**CK1**), and glycogen synthase kinase 3β (**GSK3β**) (van Loosdregt et al., 2013). When Wnt interacts with frizzled (**FZD**) receptor, it inhibits GSK3β activity, inactivates the destruction complex and increases β-catenin protein levels (Padwal et al., 2020; Staal et al., 2008). The CyclinD1 gene, a direct target of the β-catenin/LEF-1 pathway, contains a LEF-1 binding site in its promoter (Que et al., 2019). Research has shown that melatonin combines with RORα to activate the Wnt signaling pathway, which promotes the expression of CyclinD1 and enhances the growth of cashmere (Zhang et al., 2021). Furthermore, melatonin can activate GSK3β by inhibiting the phosphorylation of AKT, a serine-threonine kinase, and induce the degradation of β-catenin (Mao et al., 2012). However, it is still unknown whether the Wnt signaling pathway is involved in the melatonin nuclear receptor-mediated regulation of lymphocyte activity in response to changes in light. Therefore, this study focuses on the relationship between the nuclear receptors RORα/RORγ and the AKT/β-catenin signaling pathway in monochromatic light-induced lymphocyte proliferation in the thymus of chicks. The findings of this study aim to provide a theoretical basis for practical poultry production.

## Materials and methods

### Animal treatments

A total of 100 newly hatched broilers were purchased (Beijing Huadu Breeding Co., Beijing, China), and allocated into white light (WL, 400–700 nm, n = 15), red light (RL, 660 nm, n = 15), green light (GL, 560 nm, n = 15) and blue light (BL, 480 nm, n = 15) at randomly. Three days later, each light was divided into three groups, including the control group (n = 5), the experimental group (pinealectomy, n = 5), and sham-operated group (n = 5) (Broilers in sham-operated group underwent the same experimental operation without removing the pineal gland) . The photoperiod was 23 h of light and 1 h of darkness, the illuminance level at the bird's head was measured at 15 ± 0.3 lux. During the first week, the ambient temperature was maintained at 32 ± 2 °C and subsequently decreased by 1 °C every two days until reaching 30 °C in the second week. Throughout the experimental period, feed and water were supplied ad libitum. The diet was formulated to meet or exceed the nutrient recommendations of the National Research Council for poultry (1994).

At 14 days, 60 broilers were chosen from each treatment, then removed the left thymus of chicks aseptically. One part of the thymus preserved in low temperature (-80 °C) assessed for mRNA and protein levels by quantitative reverse transcription polymerase chain reaction (RT-qPCR) and western blotting, and the other part was fixed in 4 % paraformaldehyde in 0.1 M phosphate buffered saline (PBS, pH 7.4, 4 °C) for immunohistochemistry staining.

### Immunohistochemical staining

Immunohistochemistry staining was performed on paraformaldehyde-fixed, paraffin-embedded sections using the labeled horseradish peroxidase (HRP) method. Paraffin sections of 5 µm thick were mounted on poly-L-lysine-coated slides. The slides were de-paraffinized in xylene, rehydrated through graded alcohol. The slides were immersed in sodium citrate-hydrochloric acid buffer and boiled for 20 min (pH = 6.0), followed by slow cooling down. Endogenous peroxidase activities were blocked by 3 % H_2_O_2_ for 30 min. After nonspecific binding blocking, the slides were incubated with a rabbit against primary antibody (AKT, #9272, 1:500, CST, USA; GSK3β, 22104-1AP, 1:300, proteintech, USA; β-catenin, 51067-2-AP, 1:500, proteintech, USA), or a mouse against primary antibody (CyclinD1, abx100482, 1:200, abbexa, UK) over night. Negative controls were incubated with antibody diluent only. Slides were brought to room temperature and incubated with goat anti-rabbit secondary antibody which conjugated with biotinylated (sc-2020, 1:300, Santa Cruz, CA, USA) for 2 h at 37 °C. The sections were washed with PBS three times, added appropriate streptavidin-horseradish peroxidase into the tissue (1:300, Vector Laboratories, Burlingame, CA, USA) for 2 h. After a series of binding reactions, 3, 3-diaminobenzidine (DAB, Sigma)-H_2_O_2_ was added, and sections incubated in the dark. Finally, after a series of dehydration and hematoxylin staining, immunohistochemical staining of thymus tissue sections were completed.

### RT-qPCR

The total RNA was purified from tissue or cells of thymus using a reverse transcription kit (Thermo Fisher Scientific, Boston, USA). For each sample, 2 µg of total RNA was utilized for reverse transcription following the manufacturer's protocol. The resulting cDNA samples were stored at -20 °C.

Briefly, the PCR amplification system contained 2 μL cDNA sample, 0.4 μL target primer, 10 μL AceQ® qPCR SYBR® Green Master Mix (Vazyme, Nanjing, China), and 7.2 μL ddH_2_O; after mixing, the samples were amplified in a Roche LightCycler® 480 System (Switzerland) at 95 °C for 10 min, 95 °C for 30 s by 40 cycles, annealing at 57 °C for 30 s, and extension at 72 °C for 30 s. cGAPDH was used as internal reference for standardization. The PCR primers are listed in Table 1.

**Table 1:** Sequences of the primers used for RT-qPCR.

Genes	Primer sequences (5′-3′)	Accession no.	Product size (bp)
*RORα*	F: TGGGCATACCCCTGAAGG TA	XM_413763.2	140
	R: CCGATGCTGGTGTGTAGTCA		
*RORγ*	F: GTGGGGTAATATCGGGAGCG	XM_004948394.1	90
	R: CTTATCGGGACAACCTGCGT		
*CyclinD 1*	F: TGTCGTTCGAACCCCTCAAG	NM_205381.1	156
	R: CCATTTGCAGTAACTCGTCGG		
*GAPDH*	F: ATC ACAGCCACACAGAAGACG	NM_204305	124
	R: TGA CTTTCCCCACAGCCTTA		

F = forward primer; R = reverse primer.

### Western blot

Tissue or cells were lysed using RIPA lysis buffer, and protein quantification was conducted in accordance with the protein assay kit (CW0014, CWBIO, Beijing, China). The total protein lysate was then subjected to separation via SDS-polyacrylamide gel electrophoresis (SDS-PAGE) and subsequently transferred onto a polyvinyl difluoride (**PVDF**) membrane (Millipore, Billerica, MA, USA). After blocking the membrane with 5 % fat-free milk in TBST for 1 h at room temperature. Primary antibodies against p-AKT (#4060, 1:1000, CST, USA), AKT (#9272, 1:1000, CST, USA), p-GSK3β (PAB10055, 1:500, Abnova, Taiwan, China), GSK3β (22104-1AP, 1:500, proteintech, USA), β-catenin (51067-2-AP, 1:1000, proteintech, USA), and CyclinD1 (abx100482, 1:300, abbexa, UK) were incubated at 4 °C overnight. β-actin was used as a control (CW0096, 1:4000, CWBIO, China). HRP-conjugated anti-rabbit or anti-mouse IgG were used as secondary antibodies (goat anti-mouse IgG-HRP diluted 1: 6000; goat anti-rabbit IgG-HRP diluted 1: 6000; CoWin Biotech Co., Inc) for 2 h at room temperature. Finally, the bands were visualized by automatic chemiluminescence image analysis system (5200, Tanon, shanghai, China) and quantified by Image Analysis software (Gel-Pro Analyzer 4.5; Media Cybernetics, Rockville, MD, USA).

### Lymphocyte proliferative activity assay

At 14 days, the left thymus of broilers was taken in a sterile environment, after grinding with PBS, the cell suspension was slowly added to the chicken lymphocyte isolation solution at a ratio of 1:1 and centrifuged. Finally, the middle layer was diluted into a cell suspension with basal medium. Cell suspensions were distributed in 6-well or 96-well plates (6 × 10^6^ cells/well).The control group consisted solely of cell suspension, whereas the experimental groups received melatonin (10^-9^ M, Sigma-Aldrich, St. Louis, USA) in the presence of ConA stimulation (20 µg/mL, Sigma-Aldrich, St. Louis, USA), another group received chemical blocker for 30 min prior to the addition of ConA and melatonin. Cells were cultured at 37 °C for 44 h, and the number of viable cells was measured by adding 10 µL of MTT solution (5 mg/mL) to each well, and the cells incubated for 4 h at 37 °C. Then, the 100 μL of 10 % SDS was added to each well. The optical density was measured at 570 nm with a microplate reader (Model 680, Bio-Rad, St. Louis, MO, USA). The viability index was calculated as the experimental OD value/the control OD value. The cells in 6-well plates were extracted for mRNA and protein testing followed by 48 h.

The chemical blocker contains: 5 μM SR3335 (a selective RORα inverse agonist; MCE, New Jersey, USA), 10 μM SR1078 (a nonselective agonist of the nuclear receptor RORα and RORγ; MCE, New Jersey, USA), 1 μM GSK (an antagonist of RORγ; TargetMol, Massachusetts, USA), 5 μM LY-294 (an antagonist of PI3K; Sigma-Aldrich, St. Louis, USA), 0.5 μM HY-102 (an antagonist of AKT; MCE, New Jersey, USA), and 0.5 μM TWS119 (an antagonist of GSK3β; MCE, New Jersey, USA).

### Statistical analyses

All experimental data were analyzed by SPSS version 17.0 software (SPSS, Inc., Chicago, IL, USA) followed by Duncan's multiple rang tests. The values were expressed as mean ± SEM. The data were analyzed by performing two-way ANOVA. P-value showed ≤ 0.05 was expressed as significant statistically.

## Results

### Monochromatic light affected the expression of nuclear receptors RORα and RORγ in thymus

**Fig. 1:**
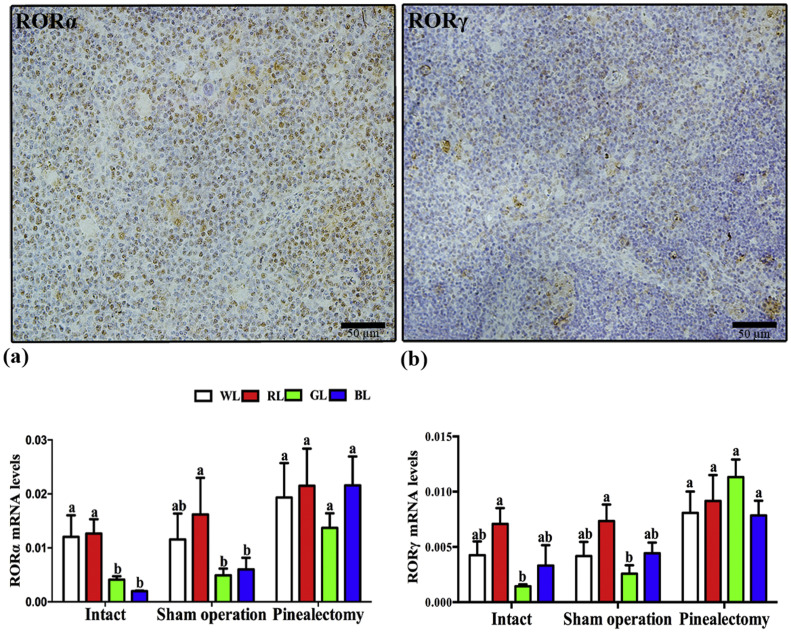
Effect of monochromatic light on the immunohistochemical positive cells and mRNA expression levels of *RORα* and *RORγ* in thymus of chicks. (a), RORα; (b), RORγ. WL, white light; RL, red light; GL, green light; BL, blue light. Data are presented as the means ± SEM. Different letters on the column indicate significant differences (*P* < 0.05) between treatment groups. Scale bar = 50 μm.

The positive expression of RORα and RORγ in thymus were observed by immunohistochemical staining. Expression of RORα and RORγ was detected in both the cortex and medulla of the thymus. The level of RORγ expression was lower than the expression of RORα (Fig. 1).

The mRNA levels of RORα and RORγ in thymus were detected by RT-qPCR (Fig. 1). The results showed that RL increased the mRNA levels of RORα by 5.36 % (*P* = 0.859) compared with WL, by 207.9 % (*P* = 0.032) compared with GL and by 536.42 % (*P =* 0.006) compared with BL in intact group. The similar results were found in sham operation group (Fig. 1a). The mRNA levels of RORγ were less than RORα in thymus, and the RL also increased RORγ levels. The mRNA levels of RORγ in RL were higher by 66.48 % (*P =* 0.178), 391.21 % (*P* = 0.013) than that of in WL and GL in intact group, respectively, also higher by 113.41 % than BL but not significantly different (*P* = 0.086). Those results were similar with the sham operation group. However, pinealectomy increased the RORα and RORγ levels by 32.90 %–259.50 % (*P* = 0.000-0.057) and 24.83 %–338.96 % (*P* = 0.000-0.062) in thymus under four light treatments compared with the corresponding sham operation group, and there were no significant differences among those lights after pinealectomy (*P* > 0.05) (Fig. 1b).

### Monochromatic light affected the expression of AKT and GSK3β in thymus

**Fig. 2:**
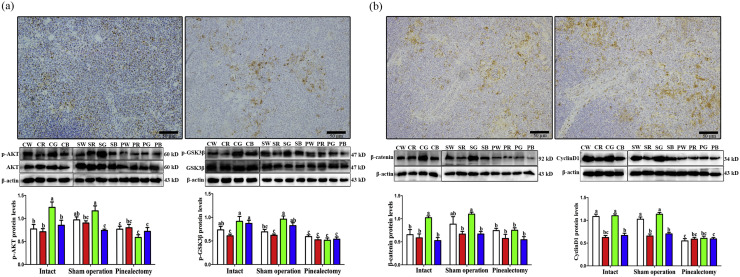
Effect of monochromatic light on the immunohistochemical positive cells and expression levels of AKT, GSK3β, β-catenin, and CyclinD1 in thymus of chicks. (a), AKT and GSK3β; (b), β-catenin and CyclinD1. WL, white light; RL, red light; GL, green light; BL, blue light. Data are presented as the means ± SEM. Different letters on the column indicate significant differences (*P* < 0.05) between treatment groups. Scale bar = 50 μm.

AKT and GSK3β were specifically located in the medulla of thymus, with no positive cells found in the cortex (Fig. 2a). Additionally, AKT-positive cells were more widely distributed in the thymic medulla. It is well-known that the thymus medulla is abundant in mature lymphocytes, suggesting that AKT/GSK3β play a crucial role in the proliferation of thymus lymphocytes.

The results of the protein levels of p-AKT and p-GSK3β under different monochromatic lights in the thymus of chicks indicated that GL increased the protein levels of p-AKT compared to WL (60.73 %, *P* = 0.003), RL (74.31 %, *P* < 0.001), and BL (45.69 %, *P* = 0.009) in the intact group. The sham operated groups showed similar results to the intact groups. However, pinealectomy decreased the p-AKT protein levels by 2.97 %–98.31 % (*P* = 0.000-0.815) compared to the corresponding monochromatic light in the sham operation, and there were no significant differences among the light treatments (*P* > 0.05, Fig. 2a).

The GL also promoted the protein levels of p-GSK3β by 5.06 % to 50.31 % (*P =* 0.008-0.675) compared with other lights in thymus, and RL decreased the protein levels of p-GSK3β. There were no significant differences between the sham operated and the intact groups (*P* > 0.05). After pinealectomy, the protein levels of p-GSK3β decreased by 17.14 %–86.79 % (*P =* 0.000-0.151) compared to the corresponding sham operation, and the significant differences disappeared among those light treatments (*P* > 0.05, Fig. 2a).

### Monochromatic light affected the expression of β-catenin and cyclinD1 in thymus

Similar to the expression of GSK3β, positive expression of β-catenin and CyclinD1 was also observed in the medulla of the thymus, which was widely distributed in the medulla. This suggests that both β-catenin and CyclinD1 are involved in T-lymphocyte proliferation in the thymus (Fig. 2b).

GL obviously increased the protein levels of β-catenin in thymus. The protein levels of β-catenin in GL were higher by 56.22 % (*P =* 0.003), 74.67 % (*P* = 0.001), and 94.24 % (*P* < 0.001) than that of in WL, RL, and BL, respectively. The GL significantly increased the expression levels of β-catenin by 24.64 %–64.72 % (*P* = 0.002-0.096) in the sham operation. However, pinealectomy decreased the protein levels of β-catenin by 16.76 %–46.49 % (*P* = 0.000-0.431). No significant differences were observed in pinealectomy group (*P* > 0.05, Fig. 2b).

The protein levels of CyclinD1 in GL were increased by 1.49 %–76.51 % (*P* = 0.000-0.803) compared to other light treatments in the intact group. However, RL significantly reduced the protein levels of CyclinD1, which was similar to that in the sham operated. Meanwhile, the protein levels of CyclinD1 decreased by 11.77 %–87.27 % (*P* = 0.000-0.266) after pinealectomy, and no significant different was detected among the various light treatments in the pinealectomy group (*P* > 0.05, Fig. 2b).

The Pearson correlation analysis showed a positive correlation between changes in the protein expression levels of thymus β-catenin and CyclinD1 (r = 0.9389, *P* < 0.0001).

### Effect of RORα and RORγ on the expression of p-AKT/β-catenin/cyclinD1

**Fig. 3:**
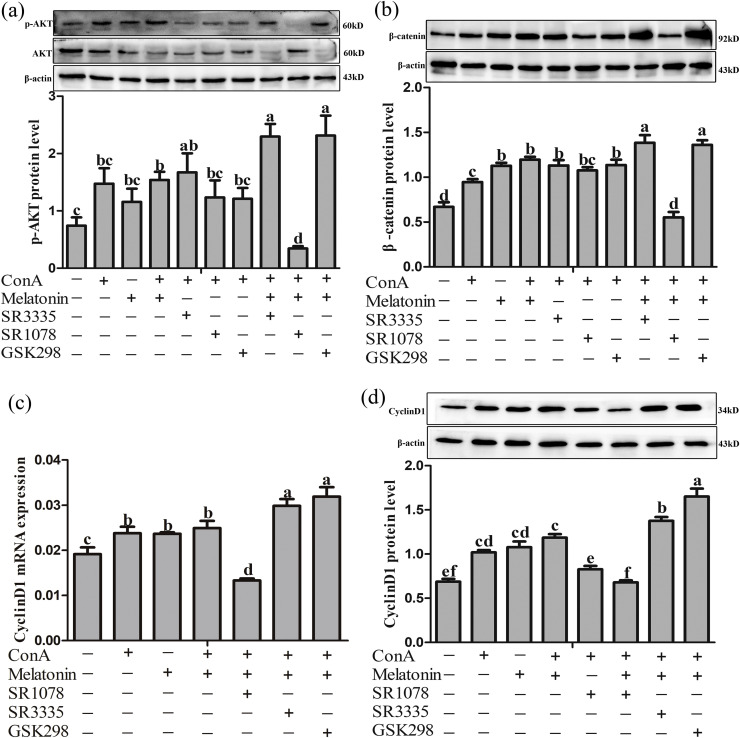
Effect of RORα and RORγ on the p-AKT/β-catenin/CyclinD1 protein expression in thymic lymphocytes. (a), Protein levels of p-AKT; (b), Protein levels of β-catenin; (c), mRNA levels of *CyclinD1*; (d), Protein levels of CyclinD1. SR3335: RORα antagonist (5 μM); SR1078: RORα and RORγ agonist (10 μM); GSK298: RORγ antagonist (1 μM). Data are presented as the means ± SEM. Different letters on the column indicate significant differences compared with the control group (*P* < 0.05).

In cultured thymic lymphocytes, the SR1078 (10 μM), SR3335 (5 μM) or GSK298 (1 μM) was added 30 min before the addition of ConA (20 μg/mL) + melatonin (10^-9^ M) group. Compared to the control group, ConA + melatonin group exhibited a significant increase in protein levels of p-AKT by 107.84 % (*P =* 0.036) in thymic lymphocytes, while a selective RORα inverse agonist (SR3335, 5 μM) under the stimulation of ConA had no effect on the expression of p-AKT (*P* > 0.05, Fig. 3a). The addition of a nonselective agonist of the nuclear receptors RORα and RORγ (SR1078, 10 μM) to the cell suspension did not alter the protein levels of p-AKT. Meanwhile, the antagonist of RORγ (GSK298, 1 μM) also did not change the expression levels of p-AKT. However, SR3335 and GSK298 obviously increased the p-AKT under the stimulation of ConA and melatonin by 49.26 % (*P* = 0.046) and 50.45 % (*P* = 0.041), while SR1078 decreased the protein levels of p-AKT by 346.35 % (*P* = 0.001) compared with ConA + melatonin group.

The protein levels of β-catenin were also examined (Fig. 3b). The addition of SR1078, SR3335, and GSK298 alone to the cultured thymic lymphocytes had no effect on the protein levels of β-catenin (*P* > 0.05). However, in comparison to the control group, the ConA + melatonin group showed an increase in β-catenin levels in thymic lymphocytes (78.61 %, *P* < 0.001). Additionally, compared to the melatonin + ConA group, the melatonin + ConA + SR3335 group exhibited a 15.82 % increase in β-catenin levels (*P* = 0.011). Similarly, the antagonist of RORγ, GSK298, also increased the protein levels of β-catenin by 13.86 % (*P* = 0.039). In contrast, a nonselective agonist of the nuclear receptors RORα and RORγ, SR1078, obviously decreased the protein levels of β-catenin by 115.99 % (*P* < 0.001).

Furthermore, the melatonin + ConA group demonstrated a significant increase in both mRNA and protein levels of CyclinD1 compared to the control group (30.16 %, *P* = 0.005; 72.34 %, *P* < 0.001; Fig. 3c and d), and these effects were further enhanced by SR3335 (19.76 %, *P* = 0.016; 16.05 %, *P* = 0.008) and GSK298 (27.88 %, *P* = 0.001; 39.13 %, *P* < 0.001). Conversely, administration of SR1078 decreased the protein levels of CyclinD1 by 74.70 % (*P* < 0.001) in T-lymphocytes.

### Effect of PI3K/AKT/β-catenin on the expression of β-catenin and cyclinD1

**Fig. 4:**
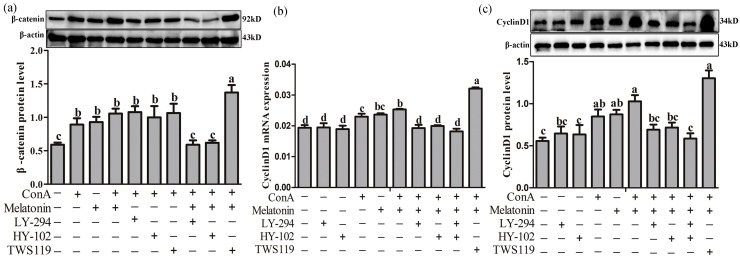
Effect of the PI3K/AKT/GSK3β on the β-catenin/CyclinD1 expression. (a), Protein levels of β-catenin; (b), mRNA levels of *CyclinD1*; (c), Protein levels of CyclinD1. LY-294 (5 μM): PI3K antagonist; HY-102 (0.5 μM): AKT antagonist; TWS119 (0.5 μM): GSK3β antagonist. Data are presented as the means ± SEM. Different letters on the column indicate significant differences compared with the control group (*P* < 0.05).

Compared with melatonin + ConA group, LY-294 and HY-102 decreased the protein levels of β-catenin by 48.41 % (*P* = 0.025) and 44.39 % (*P* = 0.008). While the GSK3β antagonist TWS119 increased the protein levels of β-catenin by 29.86 % (*P* = 0.045) compared with melatonin + ConA group (Fig. 4a). Similar to the results for β-catenin, melatonin + ConA increased the mRNA and protein levels of CyclinD1. However, both LY-294 and HY-102 decreased the mRNA and protein levels of CyclinD1 by 26.86 %-39.15 % (*P* < 0.001) and 43.45 %–75.52 % (*P* = 0.000-0.005) compared with melatonin + ConA group. The GSK3β antagonist TWS119 increased the mRNA and protein levels of CyclinD1 by 26.70 % (*P* < 0.001) in T-lymphocytes (Fig. 4b and c).

## Discussion

Many factors affect the immune function of birds, light is one of them that can't be ignored (Kernbach et al., 2018; Wu et al., 2022a). The T/B-lymphocytes proliferation of spleen from 6-week-old chickens grown in intermittent lighting were higher than those of chickens grown in constant lighting (Kliger et al., 2000). In our study, we focused on the impact of light color on lymphocyte development in chickens. Our previous results found that GL promoted lymphocyte proliferation of spleen (Zhang et al., 2014; Guo et al., 2017), thymus (Chen et al., 2016), and the bursa of Fabricius in broilers (Li et al., 2015b. Conversely, we also demonstrated that monochrome red light reduces lymphocyte activity in the chicken thymus. Furthermore, we investigated the effect of melatonin nuclear receptors on apoptosis mediated by monochrome red light by modulating downstream Wnt signaling.

In our investigation, we observed variations in melatonin levels in chicken plasma under different monochromatic lights. GL obviously increased melatonin expression, while RL decreased melatonin expression (Xiong et al., 2019). This is because the pineal gland plays an essential role in transmitting light information and regulates various endocrine functions by rhythmically secreting melatonin (Butler et al., 2010). In hamsters, the levels of the cytokine IL-2 in the serum were positively correlated with melatonin levels under different photoperiodic conditions (Vishwas and Haldar, 2013). The main way melatonin works is by binding to receptors. Both the melatonin membrane receptors MT1 (Mel 1a) and MT2 (Mel 1b) have been identified in both central and peripheral sites in mammals and birds, while the Mel1c receptor has been found specifically in birds (Reppert et al., 1995; Klosen, 2024). This indicates that melatonin receptors exhibit tissue-specificity. Research pointed out that melatonin through MT1 but not MT2 decreases biliary damage and liver fibrosis during cholestatic liver injury (Wu et al., 2022b), while melatonin exerted protective effects against hepatic fibrosis through MT2 activation (Kim and Cheon, 2024).

In this study, we examined the expression of melatonin nuclear receptors in the chicken thymus and found that the expression of nuclear receptors RORα and RORγ was prominent, whereas the expression of RORβ was challenging to detect. Similar to our findings, there are studies that RORα and RORγ are mainly involved in the regulation of the immune system, while RORβ is mainly found in the nervous system (Lardone et al., 2011; Nakagawa and O'Leary, 2003). Our previous study has identified that melatonin increased lymphocyte proliferation in the thymus by negatively regulating RORα expression under GL (Xiong et al., 2019). Studies have suggested that melatonin regulates nuclear receptors by reducing the level of RORα in cells in a time-dependent manner (Lardone et al., 2011). Additionally, melatonin may indirectly regulate nuclear receptors by binding to membrane receptors to modulate RORα expression (Jetten et al., 2001). Melatonin regulates RORα/γ expression through MT1 receptor, and promotes T cell differentiation in autoimmune diseases (Farez et al., 2015). In our previous experiment, melatonin through membrane receptor Mel1a, further increased the protein expression of nuclear receptor RORα under monochromatic red light (Xiong et al., 2020).

AKT regulates T-cell function have been proved (Rogel et al., 2017). In the study, the protein levels of p-AKT were detected under monochromatic light treatments, and it was found that GL promoted the expression of p-AKT in the thymus of chicks. Additionally, exogenous melatonin increased the protein expression of p-AKT in T-lymphocytes under GL. This finding aligns with a previous study that showed melatonin stimulated AKT phosphorylation in neuronal cells (Onphachanh et al., 2017). However, this effect of melatonin on p-AKT was blocked by RORα/γ. RORα is known to regulate the AKT signaling pathways in the context of lipid homeostasis in skeletal muscle (Raichur et al., 2010). Our result showed that melatonin can negatively regulate nuclear receptors RORα/γ and then induce an increase in AKT phosphorylation.

The number of studies about Wnt signaling and its roles in the immune system has increased in recent years (Staal et al., 2008). Deletion of Wnt-1 or Wnt-4 leads to a significant decrease in the number of thymocytes without affecting their maturation pattern (Mulroy et al., 2002). Our results showed that the protein levels of p-GSK3β (Ser 9) in thymus were increased under GL, suggesting that GL inactivated GSK3β. Exposure to light-at-night suppresses the nocturnal pineal melatonin synthesis, disrupting the circadian rhythm of GSK3β phosphorylation (Mao et al., 2012). When GSK3β activity was inhibited, the effects of melatonin on lymphocyte proliferation were decreased.

The destruction complex, which consists of GSK3β, CK1, and Axin in the cytoplasm, constantly degrades β-catenin levels. Activated GSK3β triggers the degradation of β-catenin through ubiquitination or proteasome. However, the serine-threonine kinase AKT can inhibit the activity of GSK3β (Ser 9), promoting the translocation of β-catenin to the nucleus and its dimerization with TCF/LEF to activate the transcription of Wnt target genes (van Es et al., 2003). The β-catenin protein expression in thymus was also increased in GL. In vitro, melatonin promoted the protein expression of β-catenin. Similar to our results that melatonin activates β-catenin to prevent neuronal cell death through regulating anti-apoptotic protein (Jeong et al., 2014). When GSK3β is blocked, the protein expression of β-catenin was further increased. However, the effect of melatonin on β-catenin was reversed by RORα/γ. These further suggest that melatonin can regulate the expression of Wnt signal through RORα/γ, and mediate the monochromatic light-induced lymphocyte proliferation. In vitro, the antagonist of AKT also blocked the effect of melatonin on β-catenin. Others have testified that melatonin inhibits the serine-threonine kinase AKT phosphorylation to activate GSK3β, inducing β-catenin degradation (Mao et al., 2012).

CyclinD1 is closely associated with the immune system (Bazyka et al., 2015) and can regulate the proliferation of thymus epithelial cells (Robles et al., 1996). Our findings indicate that the protein levels of CyclinD1 were elevated in the thymus of chicks exposed to GL, suggesting an enhancement in the proliferation activity of immune cells in the thymus. In vitro experiments, we also observed that melatonin increased both the mRNA and protein levels of CyclinD1. And the CyclinD1 gene has been demonstrated to be a direct target for transactivation via the β-catenin/LEF-1 binding site in the CyclinD1 promoter (Mao et al., 2012). Therefore, the increased CyclinD1 protein levels in response to melatonin treatment was regulated by β-catenin was determined. Our results demonstrated that the effect of melatonin on CyclinD1 was inhibited by nuclear receptors RORα and RORγ, but enhanced by the PI3K/AKT signal or β-catenin.

In conclusion, monochromatic red light exposure promotes the expression of RORα and RORγ in the thymus of chicks. RORα/RORγ, through the signaling molecule PI3K/p-AKT, inactivate AKT and activate GSK3β, leading to a decrease in nuclear β-catenin levels. This, in turn, suppresses the expression of CyclinD1 and influences the process of monochromatic red light on the proliferation of T-lymphocytes in the thymus of chicks.

## Conclusions

We demonstrate that melatonin nuclear receptor RORα/RORγ negatively regulate monochromatic red light induced-T-lymphocyte proliferation in the thymus, by activating the PI3K/AKT signal, then through p-GSK3β (Ser9), β-catenin was suppressed. Eventually, the expression of the Cyclin D1 gene decreased, leading to reduced cell proliferation. Overall, our paper contributes new theoretical foundations regarding the impact of light color on the proliferation activity of chicken thymus lymphocytes. We hope that further research in this area will be pursued.

## Declaration of competing interest

The authors declare that they have no known competing financial interests or personal relationships that could have appeared to influence the work reported in this paper.
